# SOCS1-Derived Peptide Administered by Eye Drops Prevents Retinal Neuroinflammation and Vascular Leakage in Experimental Diabetes

**DOI:** 10.3390/ijms20153615

**Published:** 2019-07-24

**Authors:** Cristina Hernández, Patricia Bogdanov, Carmen Gómez-Guerrero, Joel Sampedro, Cristina Solà-Adell, Carmen Espejo, Marta García-Ramírez, Ignacio Prieto, Jesús Egido, Rafael Simó

**Affiliations:** 1 Diabetes and Metabolism Research Unit, Vall d’Hebron Research Institute, Universitat Autònoma de Barcelona, 08035 Barcelona, Spain; 2 Centro de Investigación Biomédica en Red de Diabetes y Enfermedades Metabólicas Asociadas (CIBERDEM), Instituto de Salud Carlos III (ISCIII), 28029 Madrid, Spain; 3 Renal, Vascular and Diabetes Research Lab, Instituto de Investigacion Sanitaria-Fundación Jimenez Diaz (IIS-FJD), Autonoma University of Madrid (UAM), 28040 Madrid, Spain; 4 Neurology-Neuroimmunology Department, Vall d’Hebron Research Institute, 08035 Barcelona, Spain

**Keywords:** diabetic retinopathy, neuroinflammation, suppressors of cytokine signaling, microvascular impairment, db/db mouse

## Abstract

Current treatments for diabetic retinopathy (DR) target late stages when vision has already been significantly affected. Accumulating evidence suggests that neuroinflammation plays a major role in the pathogenesis of DR, resulting in the disruption of the blood-retinal barrier. Suppressors of cytokine signaling (SOCS) are cytokine-inducible proteins that function as a negative feedback loop regulating cytokine responses. On this basis, the aim of the present study was to evaluate the effect of a SOCS1-derived peptide administered by eye drops (2 weeks) on retinal neuroinflammation and early microvascular abnormalities in a db/db mouse model. In brief, we found that SOCS1-derived peptide significantly reduced glial activation and neural apoptosis induced by diabetes, as well as retinal levels of proinflammatory cytokines. Moreover, a significant improvement of electroretinogram parameters was observed, thus revealing a clear impact of the histological findings on global retinal function. Finally, SOCS1-derived peptide prevented the disruption of the blood-retinal barrier. Overall, our results suggest that topical administration of SOCS1-derived peptide is effective in preventing retinal neuroinflammation and early microvascular impairment. These findings could open up a new strategy for the treatment of early stages of DR.

## 1. Introduction

Diabetic retinopathy (DR) remains a leading cause of blindness in developed countries. At present, treatments for DR (i.e., laser photocoagulation, intravitreal injections of anti-VEGF agents or corticosteroids) are administered in advanced stages of the disease and, therefore early therapeutic strategies based on a better understanding of the pathogenesis of DR can be envisaged as a more efficient therapeutic approach [1].

DR has been classically defined as a microvascular complication of diabetes. However, a growing body of evidence indicates that retinal neurodegeneration is an early event in the pathogenesis of DR, which could be linked with the development of microvascular abnormalities [2]. A number of therapeutic strategies based on the main pathogenic mechanisms involved in neurodegeneration have been proposed [3]. Systemic administration of drugs blocking these pathways is very unlikely to reach the retina at pharmacological concentrations and, in addition, could have serious adverse effects. On the other hand, if the early stages of DR are the therapeutic target, aggressive treatments such as intravitreal injections would be unacceptable. Topical treatment with neuroprotective agents in the form of eye drops has been neglected as a possible option because of a general assumption that the posterior chamber of the eye cannot be reached by this route. However, there is emerging evidence that several peptides administered by eye drops can reach the retina in pharmacological concentrations, at least in animal models [4,5,6,7,8,9]. Apart from allowing self-administration, eye drops limit their action to the eye and minimize the associated systemic effects.

Inflammation participates in the pathogenesis of both retinal neurodegeneration and microvascular damage and, therefore, could be envisaged as a key therapeutic target in early stages of DR [10,11,12]. Among the proinflammatory cytokines involved in the pathogenesis of DR, IL-1β, IL-6, and TNF-α play a relevant role in the development of DR [13]. The main source of proinflammatory cytokines in DR is local synthesis by the retina [3], and several studies have pointed out retinal Müller glial cells and microglia as the initiators of retinal neuroinflammation [14]. Increased levels of these inflammatory mediators may lead to an early, persistent chronic inflammatory condition in the diabetic retina resulting in leukocyte activation, adhesion to the vascular endothelium (leukostasis) and disruption of the blood-retinal barrier (BRB) [15,16], the main cause of diabetic macular edema (DME). Because cytokines exert profound effects on numerous cell types and cellular processes, cytokine signals are under stringent control. Suppressors of cytokine signaling (SOCS) proteins are a family of intracellular cytokine-inducible proteins, consisting of eight members (SOCS1–7 and cytokine-inducible SH2 containing protein), SOCS1 being one of the best characterized [17]. SOCS molecules function as components of a classical negative feedback loop regulating the initiation, intensity, duration, and quality of cytokine responses, which are closely linked to the Janus family of intracellular tyrosine kinases (JAKs) and their substrates, the signal transducers and activators of transcription (STATs). SOCS1 is rapidly induced following stimulation by several cytokines, and it attenuates cytokine receptor signaling by its ability to bind and inhibit all four JAK kinases (JAK1–3 and TYK2) [18]. SOCS1 is also a key regulator of M1/M2 macrophage functions [17,18] and a crucial negative regulator of IL-6 and TNF-α, two main cytokines involved in DR. In addition, SOCS1 expression is markedly induced in retinal cells during ocular inflammation [19]. Our group previously provided direct evidence of a link between the JAK/STAT/SOCS axis and hyperglycemia-induced cell responses in the kidney [20]. In addition, we recently reported that a cell-permeable peptide mimicking the kinase-inhibitory region of the suppressor of the cytokine signaling-1 (SOCS1) regulatory protein protects against nephropathy by suppressing STAT-mediated cell responses to diabetic conditions in vitro and in vivo [21]. Since JAK/STAT also plays an essential role in the inflammatory process that occurs in the diabetic retina [22], it is reasonable to postulate that the enhancement of SOCS1 signaling could be a therapeutic target for DR. On this basis, the main aim of the present study was to evaluate the effect of a SOCS1 peptidomimetic on retinal neurodegeneration and early microvascular abnormalities in the db/db mouse, an experimental model that reproduce type 2 diabetes.

## 2. Results

Blood glucose concentrations and body weight during the 2 weeks of treatment were similar in both db/db mice treated with eye drops containing SOCS1 peptidomimetic and in db/db mice treated with eye drops containing vehicle.

### 2.1. Neuroprotective Effects of Topical Administration of SOCS1 Peptidomimetic

#### 2.1.1. Müller Glial Cells Activation

**Figure 1:**
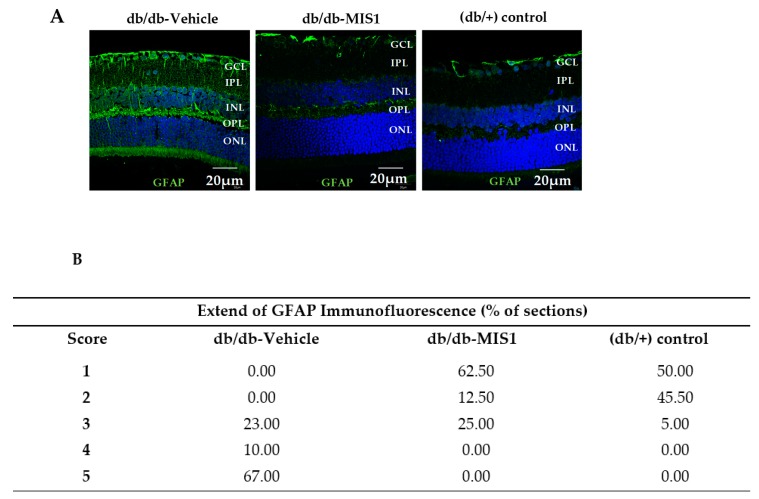
Effect of topical (eye drops) SOCS1 peptidomimetic (MIS1) administration on glial activation. (**A**) Comparison of GFAP immunoreactivity (green) in the retina among representative samples from a diabetic mouse treated with vehicle, a diabetic mouse treated with eye drops containing MIS1 and a non-diabetic mouse. Nuclei were labeled with Hoechst (blue). ONL: outer nuclear layer; INL: inner nuclear layer; GCL: ganglion cell layer. Scale bars, 20 µm. (**B**) Quantification of glial activation based on the extent of GFAP staining. N: 7 mice per group.

Glial fibrillar acidic protein (GFAP) expression was mainly confined to the retinal ganglion cell layer (GCL) in non-diabetic mice (control db/+). The diabetic mice treated with vehicle presented significantly higher GFAP expression than non-diabetic mice matched by age (Figure 1). It should be noted that 100% of diabetic mice presented a GFAP score ≥3.

The administration of the SOCS1 peptidomimetic resulted in a significant decrease of reactive gliosis, the GFAP score being ≤3 in all cases (Figure 1).

#### 2.1.2. Microglial Activation

**Figure 2:**
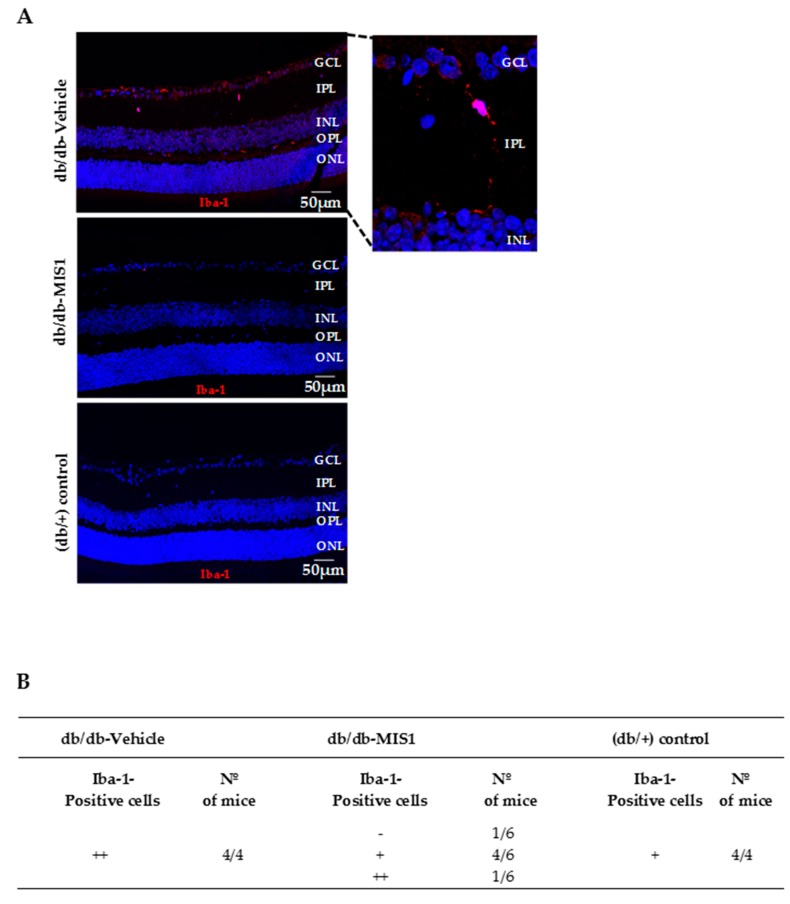
Effect of topical (eye drops) SOCS1 peptidomimetic (MIS1) administration on microglial glial activation. (**A**) Representative immunofluorescence of Iba-1 (red) in representative mice from each group. Scale bars, 50 µm. (**B**) Semiquantitative assessment of microglial activation in the retina (–: absence of positive cells for Iba-1/power field (20×); +: scattered, 1–3 cell/power field (20×); ++: moderate, 4–10 cells/ power field (20×)). N: 7 mice per group.

The presence of Iba-1 positive cells in the retina, a marker of activated microglia, was higher in retinas from diabetic mice treated with vehicle than in non-diabetic control mice. In diabetic mice treated with SOCS1 peptidomimetic the number of Iba-1 positive cells was significantly reduced in comparison with diabetic mice treated with vehicle (Figure 2).

#### 2.1.3. Apoptosis

**Figure 3:**
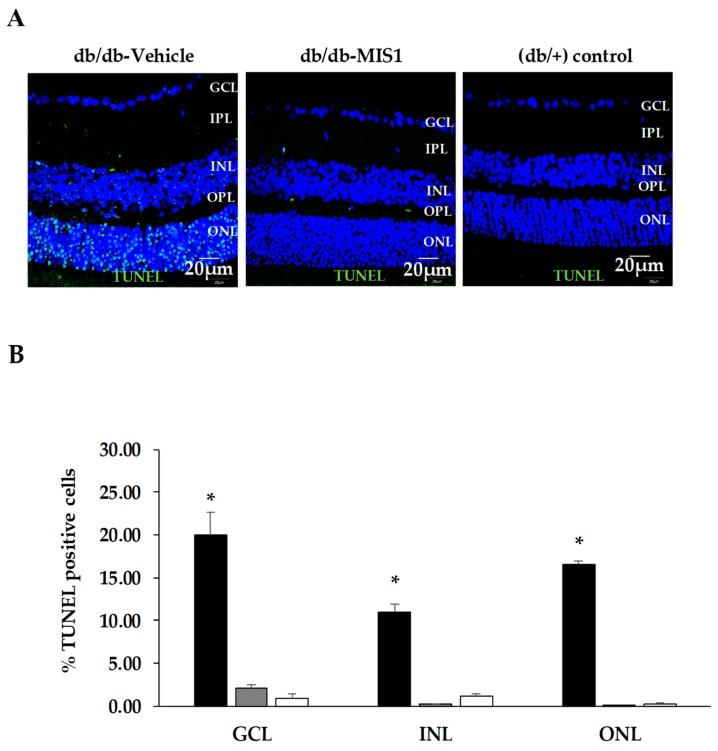
Effect of topical (eye drops) SOCS1 peptidomimetic (MIS1) administration on apoptosis. (**A**) TUNEL positive immunofluorescence (green) in a representative mouse from each group. Scale bars, 20 µm. (**B**) Percentage of TUNEL positive cells in the neuroretina. Nuclei were labeled with Hoechst (blue). ONL: outer nuclear layer; INL: inner nuclear layer; GCL: ganglion cell layer. Black columns: db/db-vehicle; Gray columns: db/db-MIS1; White columns: db/+. Results are mean ± SD. *: *p* < 0.01 in comparison with the other groups. N: 7 mice per group.

The percentage of apoptotic cells in diabetic mice was significantly higher in comparison to that observed in retinas from age-matched non-diabetic controls in all retinal layers (Figure 3).

Diabetic mice treated with SOCS1-derived peptide presented a significantly lower rate of apoptosis than diabetic mice treated with vehicle. This result was observed in all retinal layers. No differences in the percentages of apoptotic cells between diabetic mice treated with SOCS1-derived peptide and non-diabetic mice were observed.

#### 2.1.4. ERG Abnormalities

**Figure 4:**
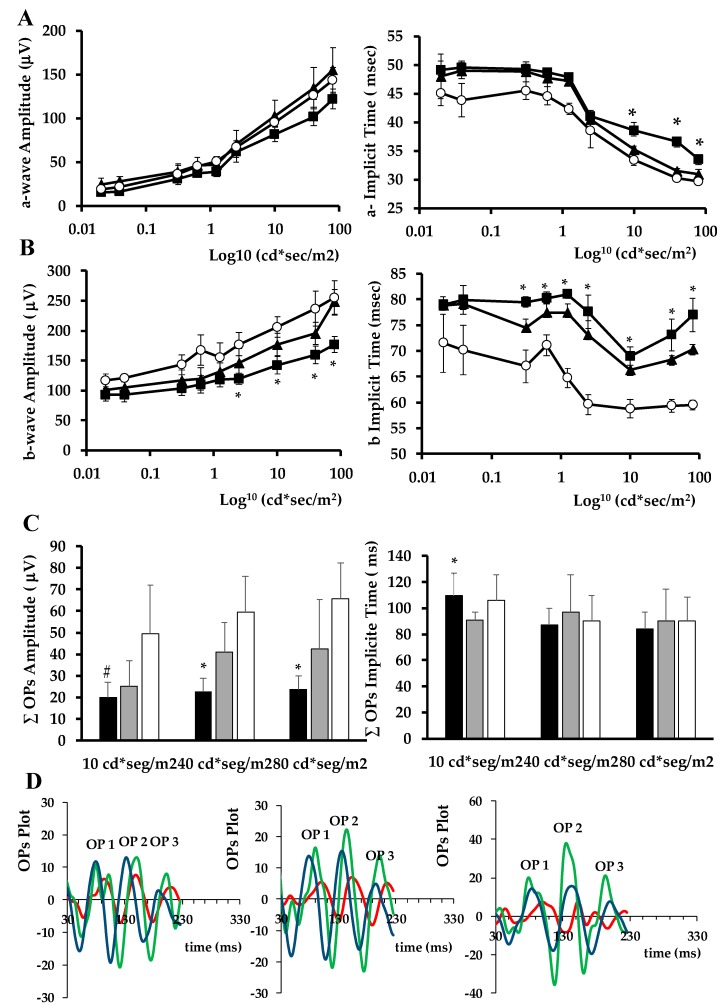
Effect of topical (eye drops) administration of SOCS1 peptidomimetic (MIS1) on ERG abnormalities. (**A**) Quantitative analyses of Amplitude and Implicit time of a-wave in db/db treated with vehicle (*n* = 10, black square), db/db treated with MIS1 (*n* = 10, black triangle) and non-diabetic mice (*n* = 10, white circle). (**B**) Amplitude and Implicit time of b-wave in db/db treated with vehicle (*n* = 10, black square), db/db with MIS1 (*n* = 10, black triangle) and non-diabetic mice (*n* = 10, white circle). (**C**) OPs Amplitude and Implicit time in the experimental groups. Results are mean ± SD. * *p* < 0.05 (db/db treated with vehicle vs. the other groups). # *p* < 0.05 (db/db treated with vehicle vs. db/db treated with MIS1). (**D**) Oscillatory potential traces in response to low, medium and high stimulus intensities (10 cd·s·m-2, 40 cd·s·m-2 and 80 cd·s·m-2) in a representative non-diabetic mouse (green), a db/db mouse treated with vehicle (red) and a db/db mouse treated with MIS1 eye-drops (blue).

The amplitude of a-wave, b-wave and oscillatory potentials (Ops) was significantly lower in diabetic mice treated with vehicle when compared with non-diabetic mice at several flash intensities (Figure 4). Concurrently, their implicit time significantly increased in diabetic mice when compared with non-diabetic mice at several flash intensities. Treatment with SOCS1 peptidomimetic was able to ameliorate these functional abnormalities induced by diabetes (Figure 4).

### 2.2. SOCS1 Treatment Prevents Vascular Leakage

**Figure 5:**
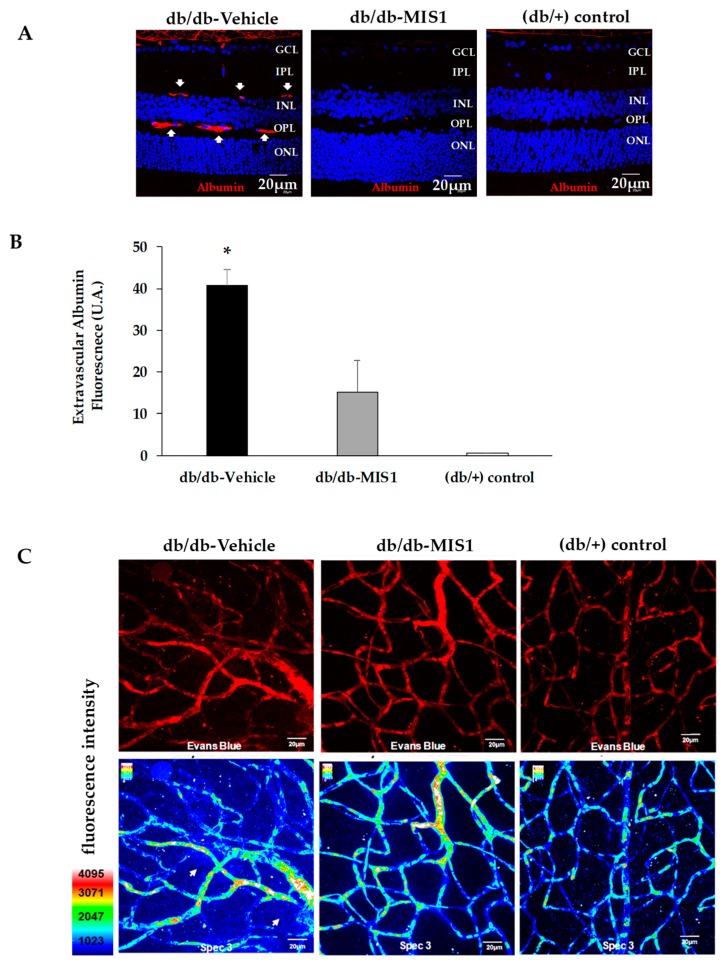
Effect of topical administration (eye drops) of SOCS1 peptidomimetic (MIS1) on vascular leakage. (**A**) Confocal microscopy images showing albumin immunofluorescence (red) in a representative diabetic mouse treated with vehicle, a diabetic mouse treated with MIS1 and a non-diabetic (db/+) mouse. Nuclei were labeled with Hoechst (blue). The presence of extravasated albumin is indicated by arrows. ONL: outer nuclear layer; OPL: outer plexiform layer; INL: inner nuclear layer; IPL: inner plexiform layer; GCL: ganglion cell layer. (**B**) Quantification of albumin immunofluorescence (*n* = 7 mice per group). AU: arbitrary units. Data are expressed as mean ± SD. * *p* < 0.05 (db/db treated with vehicle vs. the other groups). (**C**) Upper panel: Confocal immunofluorescence images of vascular permeability assessed by Evans blue dye (red) leakage in retinal whole mounts. Lower panel: Confocal immunofluorescence images with Spec3 by FV1000, Olympus. Scale bars, 20 µm. For quantification 3 mice from each group were analyzed.

Higher extravasation of albumin was observed in db/db mice treated with vehicle in comparison with non-diabetic animals. Treatment with SOCS1-derived peptide prevented albumin leakage in db/db/mice (Figure 5A,B). The beneficial effect of the topical treatment with SOCS1 peptidomimetic on vascular leakage was clearly shown by using the Evans Blue method (Figure 5C).

### 2.3. Mechanisms of Action

#### 2.3.1. Glutamate Metabolism

**Figure 6:**
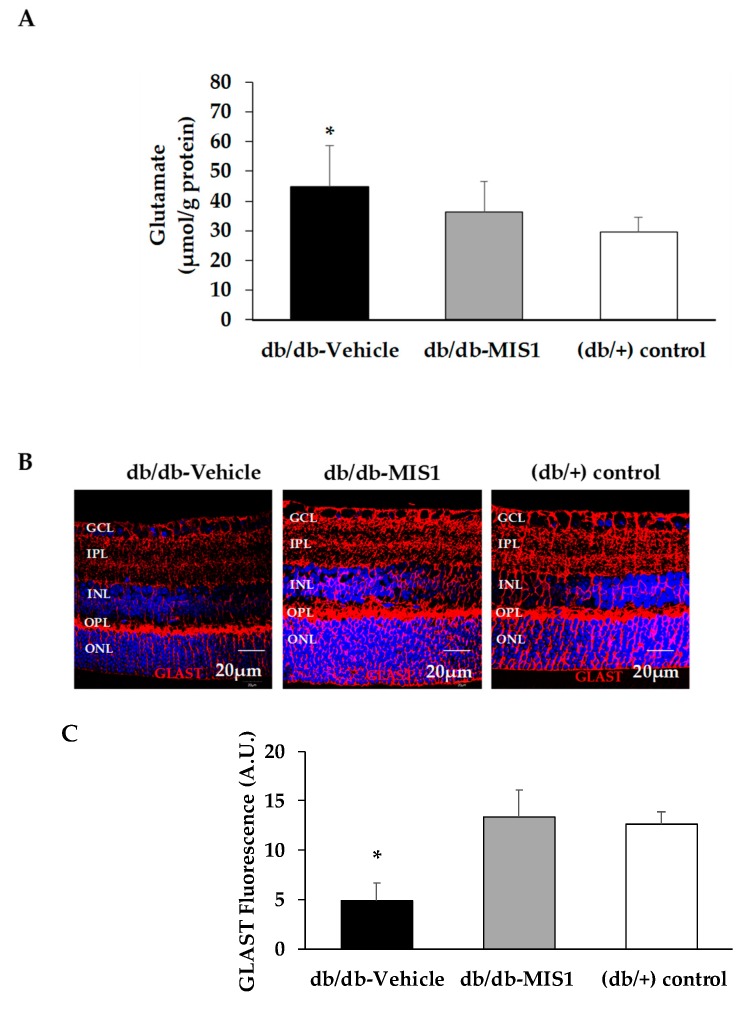
Effect of topical administration (eye drops) of SOCS1 peptidomimetic (MIS1) on glutamate and GLAST. (**A**) Retinal concentration of glutamate measured by HPLC in the experimental groups. Results are mean ± SD. * *p* < 0.01 in comparison with the control group. (**B**) Comparison of GLAST immunofluorescence (red) between representative samples from a db/db mouse treated with vehicle, a db/db mouse treated with MIS1 and a non-diabetic mouse. Nuclei were labeled with Hoechst (blue). ONL: outer nuclear layer; INL: inner nuclear layer; GCL: ganglion cell layer. (**C**) Quantification of GLAST immunofluorescence in arbitrary units (A.U). Results are mean ± SD. * *p* < 0.01 vs. the other groups.

Glutamate levels (µmol/g protein) in the diabetic retinas were higher than in the non-diabetic retinas (Figure 6A). In diabetic mice treated with SOCS1 peptidomimetic glutamate concentration was lower than in diabetic mice treated with vehicle, but without reaching statistical significance.

GLAST, the main glutamate transporter expressed by Müller cells, accounts for at least 50% of glutamate uptake in the mammalian retina [23]. GLAST content was downregulated in retinas from diabetic mice treated with vehicle, but in diabetic mice treated with SOCS1 peptidomimetic, GLAST downregulation was prevented (Figure 6B,C).

#### 2.3.2. Expression of Proinflammatory Cytokines and VEGF

**Figure 7:**
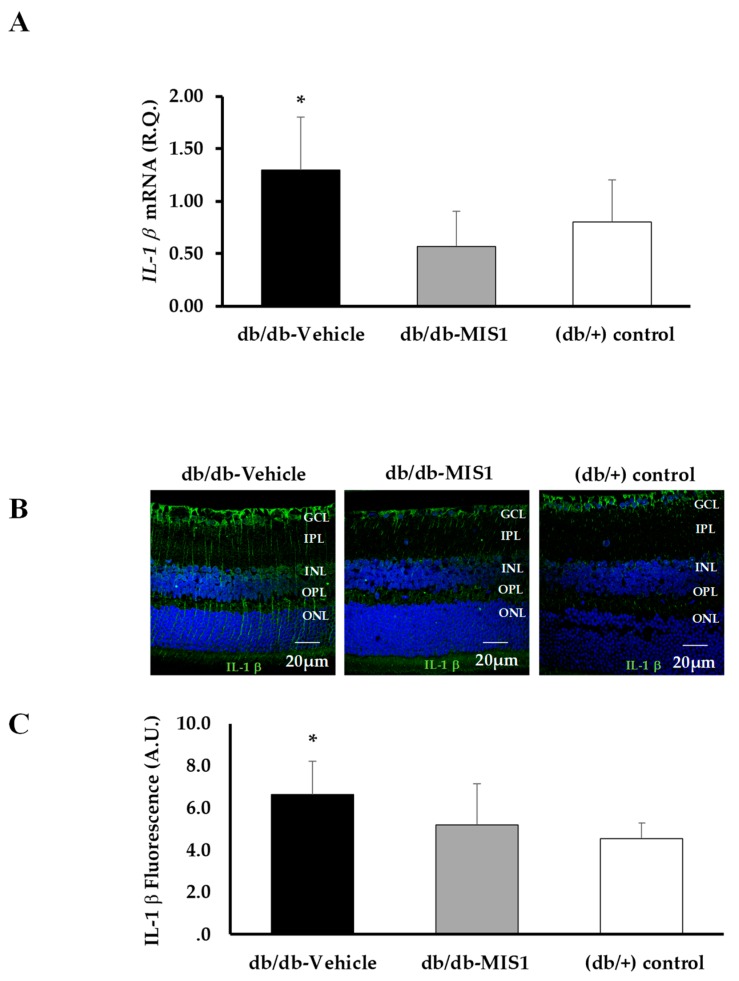
Effect of topical administration (eye drops) of SOCS1 peptidomimetic (MIS1) on IL-1β. (**A**) Quantification of IL-1β mRNA expression by RT-PCR. Results are mean ± SD. * *p* < 0.05 in comparison with the other groups. (**B**) IL-1β immunofluorescent retinal images obtained by confocal immunofluorescence from a representative db/db mouse treated with vehicle, a db/db mouse treated with MIS1 and a non-diabetic (db/+) mouse. (**C**) Quantification of IL-1β total fluorescence (*n* = 7 retinas per group). AU: arbitrary units. Data are expressed as mean ± SD. ONL: outer nuclear layer; OPL: outer plexiform layer; INL: inner nuclear layer; IPL: inner plexiform layer; GCL: ganglion cell layer.

SOCS1 peptidomimetic treatment was able to prevent the overexpression (mRNA and protein) of IL-1β induced by diabetes (Figure 7A–C).

**Figure 8:**
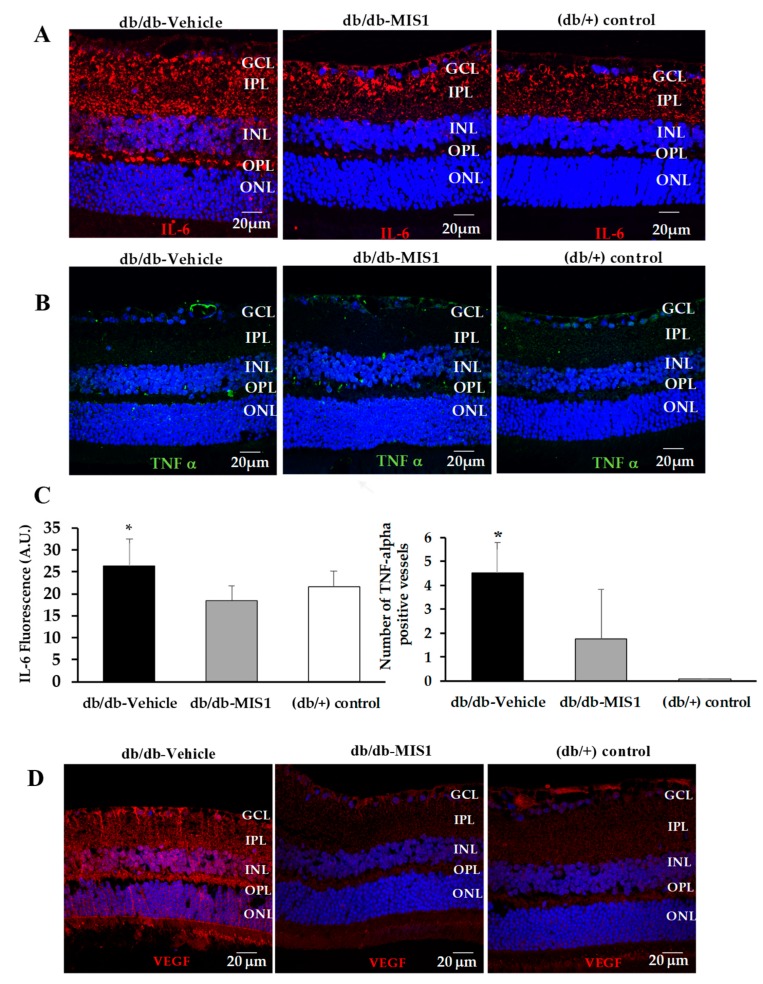
Effect of topical administration (eye drops) of SOCS1 peptidomimetic (MIS1) on IL-6 (**A**,**C**), TNF-α (**B**,**C**) and VEGF (**D**). *n* = 7 retinas per group. AU: arbitrary units. Data are expressed as mean ± SD. * *p* < 0.05 in comparison with the other groups. ONL: outer nuclear layer; OPL: outer plexiform layer; INL: inner nuclear layer; IPL: inner plexiform layer; GCL: ganglion cell layer.

Although the reduction in mRNA levels of TNF-α and IL-6 after MIS1 treatment was non-statistically significant, a significant reduction of IL-6 content and perivascular TNF-α deposition was observed in mice treated with SOCS1 peptidomimetic in comparison with vehicle (Figure 8A–C).

Regarding VEGF, SOCS1 peptidomimetic treatment significantly decreased the overexpression of VEGF induced by diabetes (Figure 8D).

#### 2.3.3. SOCS1 Peptide Suppresses STAT Activation and Downstream Gene Expression in Human Retinal Pigment Epithelial Cells

**Figure 9:**
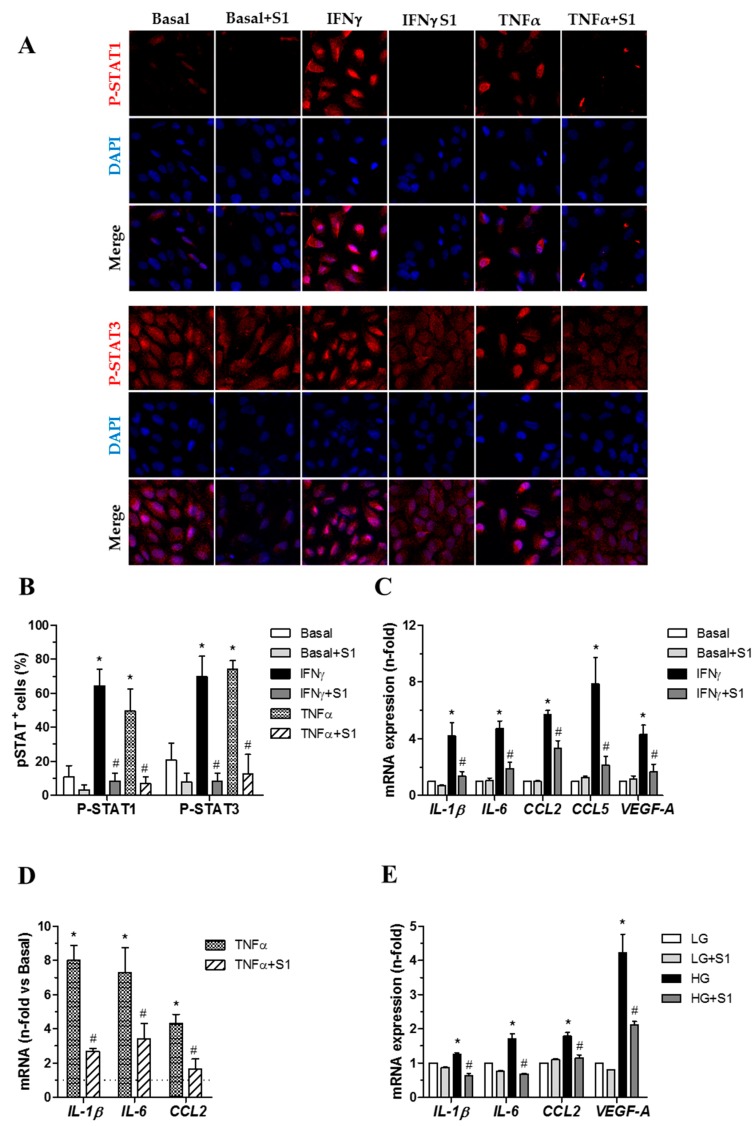
Effects of SOCS1 peptidomimetic peptide in vitro. (**A**) Representative confocal images of p-STAT1 and p-STAT3 localization in ARPE-19 cells under control conditions and after 30 min of stimulation with IFN ϒ (10 ng/mL) and TNFα (10 ng/mL) in the presence or absence of SOCS1 peptidomimetic (S1, 100 μg/mL). (**B**) Quantification of p-STAT-positive cells per total cells (DAPI-positive nuclei). (**C**–**E**) Real-time PCR analysis of indicated genes in ARPE-19 cells pretreated with SOCS1 peptide and stimulated with IFN ϒ (**C**), TNFα (**D**) and high-glucose (HG; **E**). Values normalized by 18S are expressed as fold increases over basal (dashed line in **D**) and low-glucose (LG) conditions. Bars represent the mean ± SEM of duplicate determinations from 4 experiments. * *p* < 0.05 vs. Control, # *p* < 0.05 vs. Stimulation.

To confirm the in vivo results, we analyzed the impact of SOCS1 peptide in ARPE-19 cells exposed to conditions mimicking the diabetic milieu. Confocal microscopy studies revealed that SOCS1 peptide prevented the phosphorylation and nuclear translocation of STAT1 and STAT3 proteins induced by IFNγ (Figure 9A) and TNFα (Figure 9A). Real-time PCR demonstrated that ARPE-19 cells responded to either cytokines or hyperglycemia by upregulating proinflammatory genes such as cytokines (IL-1β and IL-6) and chemokines (CCL2, CCL5) as well as VEGF, an established inducer of vascular leakage (Figure 9C–E). Furthermore, pretreatment of cells with SOCS1 peptide resulted in a significant reduction in the mRNA expression of those genes (Figure 9C–E).

## 3. Discussion

In the present study we provide evidence that a SOCS1 peptidomimetic, administered by eye drops, has dual beneficial effects in terms of neuroprotection and the inhibition of vascular leakage. The neuroprotective action consisted of a significant reduction of both glial (macroglia and microglia) activation and apoptosis. Moreover, a significant improvement of electroretinogram (ERG) parameters was observed, thus revealing the clear impact of the histological findings on global retinal function. The inhibition of vascular leakage was clearly demonstrated by the SOCS1 peptidomimetic because the disruption of the BRB, which is an essential step in the development of diabetic macular edema, was prevented.

Many proinflammatory cytokines such as IL-1β, IL-6, IL-8, MCP-1, and TNF-α are upregulated in the diabetic retina [12,13]. It should be noted that the mean levels of IL-8 and MCP-1 within the vitreous fluid of diabetic patients have been found in the same range as those reported in pleural effusions of patients with pneumonia or tuberculosis and they correlated with proliferative DR (PDR) activity [24]. In addition, the increased vitreous levels of IL-6 and IL-8 correlated with the progression of PDR and the outcome of vitreous-retinal surgery [25]. Furthermore, the activation of the JAK/STAT signaling pathway by means of proinflammatory cytokines can lead to angiogenesis either by triggering angiogenic factors, such as VEGF, or through alternative pathways [26,27,28]. These findings underscore inflammation as crucial in the pathogenic events that lead to both early and advanced stages of DR.

The present results define a hitherto unknown therapeutic effect of SOCS1 on the diabetic retina, and are in good agreement with our previous reports on the efficacy of SOCS1 to prevent the onset and progression of diabetic nephropathy, a microvascular complication of diabetes that shares common pathogenic mechanisms with DR [20,21]. Thus, we found that intrarenal delivery of adenovirus expressing SOCS1 to diabetic rats significantly improved renal function and reduced renal lesions associated with diabetes, such as mesangial expansion, fibrosis, and the influx of macrophages [20]. Furthermore, we also demonstrated that the intraperitoneal administration of the SOCS1 peptidomimetic herein tested in an experimental mouse model of diabetes ameliorated STAT activity and resulted in reduced serum creatinine levels, albuminuria, and improvement of renal histologic changes over time. Notably, mice treated with the SOCS1 peptidomimetic also exhibited reduced leukocyte levels in the kidney and decreased expression levels of proinflammatory and profibrotic markers that were independent of glycemic and lipid changes [21].

SOCS1 inhibits lymphocyte recruitment into the retina and rats and mice with targeted over-expression of SOCS1 in the retina are partially protected from experimental autoimmune uveitis [29]. Furthermore, it has been shown that topical administration of a SOCS1 peptidomimetic suppresses uveitis and confers protection from ocular pathology during experimental autoimmune uveitis [30]. These beneficial effects were produced through direct inhibition of immunopathology mediated by T lymphocytes and by protecting resident ocular cells from apoptosis during chronic intraocular inflammation [31].

As commented, inflammation plays a major role in the pathogenesis of DR [3,10,11,12]. In fact, corticosteroids (intravitreal injections or implants) are indicated in advanced stages of DME. However, when the early stages of DR are the therapeutic target, it would be inconceivable to recommend an aggressive treatment such as intravitreal injections. In this regard, it has been reported that topical administration of corticosteroids and NSAIDs could be useful for treating DME [32,33]. However, a lack of effect in reducing retinal thickness after one year of topical administration of nepafenac was also reported [34].

Besides being a non-invasive route, the advantage of topical administration is that it ensures low systemic absorption, thus minimizing the associated systemic effects. In this regard, the negligible level of systemic absorption of a SOCS1 peptide after its topical administration has been demonstrated by the absence of inhibitory effects on peripheral immune responses [31].

Regarding the mechanisms involved in the beneficial effect of the SOCS1 peptidomimetic, we found that it reduces the upregulation of IL-1β, IL-6, TNF-α, and VEGF induced by diabetes. It should be noted that IL-1β is a pivotal inflammatory cytokine because it is able to activate nuclear factor-κB, the transcription factor which in turn governs the production of other proinflammatory cytokines [35] and plays a significant role in the degeneration of retinal capillaries induced by diabetes [36]. TNF-α downregulates tight junction proteins of endothelial cells and it is also required for VEGF-induced leakage, thus participating in the breakdown of the BRB, which is the main pathogenic factor of DME [37]. We found that TNF-α expression in diabetic mice treated with vehicle was mainly detected in the perivascular areas of the inner retina. Supporting this finding, it has been reported that in the early stages of DR, advanced glucose end products (AGEs) stimulate perivascular microglial cells to produce TNF-α [38]. It has recently been described how TNFα alone induces small-molecule permeability of the BRB in vitro, whereas the combination of TNFα, IL-1β, and VEGF induces permeability to large molecules [39]. In addition, we found that SOCS1 peptidomimetic suppressed STAT activation in RPE cells, thus downregulating the expression of IL-1β, IL-6, CCL2, and CCL5, as well as the expression of VEGF. The combination of anti-inflammatory and anti-VEGF action of SOCS1 peptidomimetic observed in the present study is an extra-value in targeting two essential events in the pathogenesis of DR. However, the design of our study does not permit us to know whether the effect on VEGF is directly mediated by the peptidomimetic SOCS1 or by the downregulation of proinflammatory cytokines.

The extracellular accumulation of glutamate plays a key role in the excitotoxicity, which plays a crucial role in neuron death induced by diabetes [2]. GLAST is the main transporter of extracellular glutamate and, therefore, GLAST downregulation induced by diabetes results in high extracellular glutamate levels, thus contributing to neurodegeneration [2]. In the present study, we provide evidence that SOCS1 avoids the increase in retinal glutamate levels and that this is associated with a dramatic inhibition of diabetes induced GLAST downregulation. Therefore, the preservation of GLAST seems one of the main mechanisms by which SOCS1 peptidomimetic decreases the levels of extracellular glutamate. It is worth mentioning that we found that a GLAST mediated reduction of glutamate accumulation is a common mechanism of drugs with associated anti-inflammatory activity [5,6,8,40]. In this regard, inflammatory cytokines such us TNF-α have been shown to interact with glutamate pathways in several important ways, including decreasing the expression of glutamate transporters on relevant glial elements and increasing the release of glutamate from astrocytes in the brain [41]. Therefore, the inhibition of TNF-α induced by SOCS1 peptidomimetic could also participate in reducing extracellular glutamate levels. However, specific experiments to confirm this hypothesis are needed.

The primary neuroinflammatory action of SOCS1 peptidomimetic suggests that it could be more effective when inflammation is a predominant event in the pathogenesis of DR. Recently there has been an increasing interest in the determination and validation of non-invasive imaging retinal parameters, as possible biomarkers of a local retinal “inflammatory condition” in DR and DME [42]. The most important imaging modalities have been spectral domain (SD)-OCT and fundus autofluorescence (FA). These imaging biomarkers include: subfoveal neuroretinal detachment and hyperreflective retinal spots/foci evaluated on SD-OCT, and foveal hyperautofluorescence evaluated on FA. These methods could be useful for identifying a subset of the diabetic population in which inflammation plays an essential pathogenic role in DR. In addition, they could be useful in prognosis and in predicting treatment response. This is important in the era of personalized or precision medicine.

The present study has several limiting factors such as the lack of dose-efficacy studies, formulation stability analyses, and specific assessments addressed to evaluate potential ocular and systemic toxicity. Therefore, further research aimed at evaluating these important issues is needed. In conclusion, our results suggest that blocking retinal neuroinflammation by topical administration of SOCS1-derived peptide could be a new strategy for the treatment of DR. However, specific clinical trials aimed at evaluating its safety and effectiveness are needed.

## 4. Materials and Methods

### 4.1. Animals

The neuroprotective effect of SOCS1 peptidomimetic was tested in a db/db mouse model. This mouse carries a mutation in the leptin receptor gene and is a model for obesity-induced type 2 diabetes. Our group previously reported that the db/db mouse reproduces the features of the neurodegenerative process that occurs in the human diabetic eye [26] and has been used for testing the capacity of several drugs in reducing neuroinflammation and vascular leakage [6,8,40].

A total of 20 male db/db (BKS.Cg-Dock7m +/+ Leprdb/J) mice aged 8 weeks were purchased from Charles River Laboratories, Inc. In addition, 10 non-diabetic db/+ (BKS.Cg-Dock7m + Leprdb/+) mice matched by age were used as a control group. The animals were randomly housed under tight environmental conditions of humidity (60%), temperature (20 °C) and cycles of 12 h/12 h light/darkness. They had free access to filtered water and “ad libitum” food.

SOCS1 peptidomimetic (10 mg/mL; 5 µL twice/daily) (*n* = 10) or vehicle (PBS; 5 µL twice/daily) (*n* = 10) eye drops were administered directly onto the superior corneal surface of each eye using a micropipette in 8 weeks-old mice. Ten non-diabetic mice matched by age served as the control group. The treatment (MIS1 or vehicle) was administered twice daily for 15 days. On day 15, the drop of MIS1 or vehicle was administered approximately two hours prior to necropsy. Mice were euthanized by cervical dislocation and the eyes were immediately enucleated. The dose of SOCS1 was selected based on our previous experience using the intraperitoneal route for treating diabetic nephropathy [21].

For the Evans Blue assay, a set of animals (*n* = 3 per group) were intravenously injected in the tail with a solution of Evans Blue (E2129 SIGMA) (17 mg/Kg body weight, in concentration 5 mg/mL dissolved in saline solution sterile). Immediately after injection, the animals visibly turned blue, confirming dye uptake and distribution. After 120 min, the mice were euthanized by cervical dislocation and the eyes were enucleated. The retinas of each animal were isolated, weighed and rapidly protected from light. Flat-mounted slides were obtained, and cover slipped with a drop of mounting medium Prolong Gold antifade reagent (Invitrogen, Carlsbad, CA, USA; Thermo Fisher Scientific, Waltham, MA, USA). Digital images from different random fields of all retinas were acquired using a confocal laser scanning microscope (FV1000; Olympus, Hamburg, Germany) at ×20 using the 561-nm laser line, and each image was recorded with identical beam intensity at the size of 1024 pixels × 1024 pixels. For quantitative analysis of the albumin-bound Evans Blue, Z-stacks retinal images (step size 1.16 µm) of different regions of the vascular tree were acquired. To remove the eye artefacts caused by the sample procedures, projections of the middle Z-plane images were analyzed with the magic wand tool (tolerance 120, eight pixels connected) of Fiji software (https://fiji.sc).

This study was approved by the Animal Care and Use Committee of VHIR (Vall d’Hebron Research Institute, CEEA 75/15, September 2015)). All the experiments were performed in accordance with the tenets of the European Community (86/609/CEE) and ARVO (Association for Research in Vision and Ophthalmology).

### 4.2. SOCS1 Derived Peptide (MIS1)

The cell-permeable MIS1 peptide containing the kinase inhibitory region of mouse SOCS1 (residues 53–68) was synthesized in solid phase using the 9-fluorenylmethoxycarbonyl strategy (Proteogenix, ProteoGenix, Schiltigheim, France), then dissolved in 1% DMSO in saline solution, filter-sterilized and stored at −80 °C until use.

### 4.3. Electroretinogram

Full field electroretinography (ERG) recordings were measured using the Ganzfeld ERG platform (Phoenix Research Laboratories, Pleasanton, CA, USA) following ISCEV (International Society for Clinical Electrophysiology of Vision) recommendations [43]. Three ERG components were assessed in terms of amplitude and timing: a-wave, b-wave, and oscillatory potentials (OPs). We added up OPs amplitudes (ƩOPs amplitude) and implicit time (ƩOPs implicit time) for the first 5 OPs.

### 4.4. Immunofluorescence Analysis

For immunohistochemical analysis, animals were intraperitoneally injected with 0.2 mL of anesthesia (proportion of solution was 1 mL ketamine and 0.3 mL xylazine) and transcardially perfused with p-formaldehyde 4%. The ocular globes were immediately enucleated, fixed in p-formaldehyde 4% for 6 h and embedded in paraffin blocks. The paraffin blocks were cut along the eye axis with a microtome and the sections of 4 µm were mounted on positive charged slides and stored at 4 °C. The sections used for immunofluorescence analyses (6 sections per retina) were obtained from central retina. Paraffin-embedded sections were deparaffinized in xylene and rehydrated in a graded ethanol series. The sections were fixed in ice-cold acid methanol for 1 minute and washed with 0.01 M phosphate buffered saline (PBS) at pH 7.4. Subsequently, antigen retrieval was performed. The sections were immersed in an antigen retrieval solution (sodium citrate 10 mM, pH 6.0) and heated in a pressure cooker at 150 °C for 4 min. They were blocked by the corresponding blocking solution for 1 h at room temperature and were incubated overnight at 4 °C with the corresponding primary antibodies described in Table 1. Next day, they were washed twice in PBS, and incubated for 1 h in darkness at room temperature along with secondary antibodies sections (Alexa 488 or Alexa 594; 1:600, Molecular Probes (Eugene, OR, USA). The sections were washed in PBS, counterstained with Hoechst (1:500) and coverslipped using a mounting solution (Prolong Gold antifade reagent Invitrogen, Thermo Fisher Scientific). Images were acquired with a confocal laser scanning microscope (FV1000; Olympus). Five fields (three corresponding to the central and two to the peripheral retina) from each section were analyzed. The same locations and number of fields were measured in all retinas. The fluorescence intensity of the images was quantified by ImageJ.

#### 4.4.1. Analysis for Glial Activation

Glial activation was evaluated by fluorescence microscopy using specific antibodies against GFAP (Glial fibrillar acidic protein). To evaluate the degree of glial activation, we used a scoring system based on the extent of GFAP staining [44] and previously used by our group [26] (Table 2).

Microglial activation was evaluated based on Iba-1 staining and analyzed by a semiquantitative score. Semiquantitative assessment of microglial activation in the retina: (–) absence of positive cells for Iba-1/power field (20×); (+) scattered, 1–3 cell/power field (20x); (++) moderate, 4–10 cells/ power field (20×).

#### 4.4.2. Apoptosis Assessment

Apoptosis was evaluated using the TUNEL (Terminal Transferase dUTP Nick-End Labeling) Dead-end Fluorometric System method (Promega Corporation, Madison, WI, USA). Briefly, sections of retina were permeabilized by incubation at room temperature for 1 min with 20 µg/mL Proteinase K solution, freshly prepared. The sections were washed in PBS, counterstained with Hoechst (1:500) and cover slipped using a mounting solution (Prolong Gold antifade reagent Invitrogen, Thermo Fisher Scientific). Apoptotic cells were identified by green fluorescein -12-dUTP -labeled DNA with 488 nm as excitation wavelength and the range for detecting positive cells in the confocal laser scanning microscope was 515–565 nm (green). Lastly, the number of green positive cells and total cells (in blue) was counted.

### 4.5. Measurement of Retinal Vascular Permeability

Retinal vascular permeability was examined by assessing the albumin leakage from blood vessels into the retina by both albumin immunofluorescence analyses and the Evans blue-albumin method (ex-vivo). The Evans blue-albumin method was used as previously described with some modifications [45,46,47]. Digital images from different random fields of all retina were acquired with a confocal laser scanning microscope (FV1000, Olympus. Hamburg, Germany) at 20× using the 561 nm laser line and each image was recorded with identical beam intensity at a size of 1024 × 1024 pixels.

### 4.6. Glutamate Quantification

Quantification of glutamate was performed by reverse phase ultra-performance liquid chromatography (UPLC) (Acquity-UPLC, Waters, Milford, MA, USA) as aminoquinoline derivatives (AccQTag chemistry, Mass Trak AAA method and instruments, Waters), following the methodology previously described by Narayan et al. [48].

### 4.7. Measurements of Cytokine Expression

Total RNAs were extracted from retinas using Trizol reagent (Invitrogen) according to the manufacturer’s instructions. The quality and concentration of RNAs were determined with a Nanochip (Agilent Technologies, Madrid, Spain) and a Nanodrop ND-1000 spectrophotometer (Thermo Fisher Scientific). cDNA reactions were performed in a 2720 Thermal Cycler with High Capacity kit reagents (Applied Biosystems, Foster City, CA, USA). Real-Time PCRs were carried out using SYBR Green PCR Master Mix (Applied Biosystems) in the 7.900 HT Sequence Detection System with 384-well optical plates. Each sample was assayed in duplicate, and negative controls were included.

Specific mouse primers for IL-1β were: forward 5′-GCAACTGTTCCTGAACTCAACT-3′ and reverse 5′-ATCTTTTGGGGTCCGTCAACT-3′. ABI SDS 2.0 RQ software and the 2−∆∆*C*_t_ analysis method were used for relative quantification (R.Q.) calculation with mouse β-actin as endogenous control (Forward: 5′-CTAAGGCCAACCGTGAAAG-3′ and reverse 5′-ACCAGAGGCATAC AGGGACA-3′).

### 4.8. In Vitro Studies

Adult Retinal Pigment Epithelial cell line-19 (ARPE-19; kindly provided by S. de Pascual-Teresa, ICTAN-CSIC, Madrid, Spain) were cultured in DMEM:F12 (1:1) medium containing 10% fetal bovine serum (FBS), 100 U/mL penicillin, 100 µg/mL streptomycin and 2 mmol/L l-glutamine (Life Technologies, Rockville, MD, USA). Serum-starved cells were pretreated for 90 min with SOCS1 peptide (100 µg/mL) before stimulation with either interferon-ϒ (IFNγ 10 ng/mL) or tumor necrosis factor-α (TNFα 10 ng/mL) for 180 min. In other experiments, cells were maintained in medium containing low-glucose (5 mmol/L d-glucose), then pretreated with SOCS1 peptide and cultured for an additional 48 h under high-glucose conditions (30 mmol/L d-glucose).

Total RNA was extracted with Tryzol (Life Technologies). Gene expression levels were analyzed by real-time quantitative PCR using Taqman gene expression assays (Il1β-Hs01555410, Il6-Hs00174131, Ccl2-Hs00234140, Ccl5-Hs00174575 and Vegf-a-Hs00900055; Applied Biosystem) and normalized to 18S housekeeping gene.

For immunofluorescence analysis, cells were seeded at 104/well onto glass coverslips. After stimulation, cells were subsequently fixed in 4% paraformaldehyde, permeabilized in 0.1% sodium citrate/0.1% Triton X-100 for 5 min and blocked for 1 h in 2% bovine serum albumin and 6% host serum. Then cells were incubated with primary antibodies against phosphorylated STATs (P-STAT1, Tyr701, 1:250, Invitrogen; P-STAT3, Ser727, 1:250, Invitrogen) followed by conjugated secondary antibodies (Alexa Fluor 568; Invitrogen) and nuclear counterstaining (4’,6-diamidino-2-phenylindole; Sigma-Aldrich, Madrid, Spain). Images were captured using a confocal fluorescent microscope (Leica, Barcelona, Spain).

### 4.9. Statistical Analysis

The results are expressed as mean ± SD. Statistical comparisons were performed with Student′s unpaired *t* test. When multiple comparisons were performed, one-way ANOVA followed by Bonferroni’s test was used. The Fisher’s exact test was used to analyze categorical variables. Levels of statistical significance were set at *p* <0.05.

## Figures and Tables

**Table 1:** Targets, dilution, blocking conditions and sources of applied primary antibodies in immunofluorescence.

Target Molecule	Clone	Blocking Conditions	Dilution	Manufacturer
GFAP	Rabbit polyclonal	10% NGS, 1% BSA in PBS pH 7.4	1/200	Abcam (ab7260)
Iba-1	Rabbit polyclonal	5% NGS, 0.1% Triton X-100 in PBS pH 7.4	1/100	Wako (019-19741)
GLAST (EAAT1)	Rabbit polyclonal	5% NGS, 0.3% Triton X-100, 3% BSA in PBS pH 7.4	1/100	Abcam (ab416)
IL-1β	Rabbit polyclonal	5% NGS, 0.1% Triton X-100, 1% BSA in PBS pH 7.4	1/100	Abcam (ab9722)
IL-6	Rabbit polyclonal	10% NGS, 0.3% Triton X-100, 3% BSA in PBS pH 7.4	1/200	Abcam (ab6671)
TNF-α	Mouse monoclonal	10% NGS, 0.1% Triton X-100,1% BSA in PBS pH 7.4	1/100	Abcam (ab8348)
VEGF	Rabbit polyclonal	10% NGS, 0.1% Triton X-100,1% BSA in PBS pH 7.4	1/200	Abcam (ab46154)
Serum Albumin	Sheep polyclonal	2.5% non-fat milk in PBS pH 7.4	1/ 500	Abcam (ab8940)

NGS: Normal goat serum; BSA: Bovine serum albumin; PBS: Phosphate-buffered saline.

**Table 2:** Scoring system based on the extent of GFAP staining.

GFAP Score	Description
1	Müller cell endfet region/GCL only
2	Müller cell endfeet region/GCL plus a few proximal processes
3	Müller cell endfeet plus many processes, but not extending to ONL
4	Müller cell endfeet plus processes throughout with some in the ONL
5	Müller cell endfeet plus lots of dark processes from GCL to outer margin of ONL

## Abbreviations

AGEs	Advanced glucose end products
BRB	Blood-retinal barrier
DME	Diabetic macular edema
DR	Diabetic retinopathy
ERG	Electroretinogram
GCL	Ganglion cell layer
GFAP	Glial fibrillar acidic protein
GLAST	Glutamate-aspartate transporter
JAKs	Janus family of intracellular tyrosine kinases
INL	Inner nuclear layer
MIS1	SOCS1 peptidomimetic
ONL	Outer nuclear layer
OPs	Oscillatory potentials
SOCS	Suppressors of cytokine signaling
STATs	Signal transducers and activators of transcription
TUNEL	Terminal Transferase dUTP Nick-End Labeling
VEGF	Vascular endothelial growth factor
